# Correction to “Nanostructured Sulfur-Doped Carbon from Biomass and Its Layer-by-Layer Self-Assembly for High-Performance Supercapacitor Electrodes”

**DOI:** 10.1021/acssusresmgt.5c00386

**Published:** 2025-09-01

**Authors:** Glaydson Simoes dos Reis, Artem Iakunkov, Jyoti Shakya, Dhirendra Sahoo, Alejandro Grimm, Helinando Pequeno de Oliveira, Jyri-Pekka Mikkola, Emma M. Björk, Mahiar Max Hamedi

Corrected versions of panels c–f of Figure 1 appear below.

**Figure 1:**
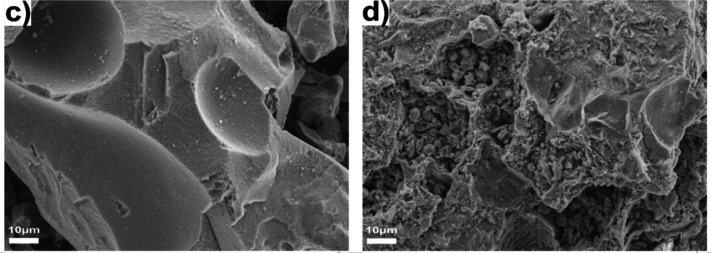

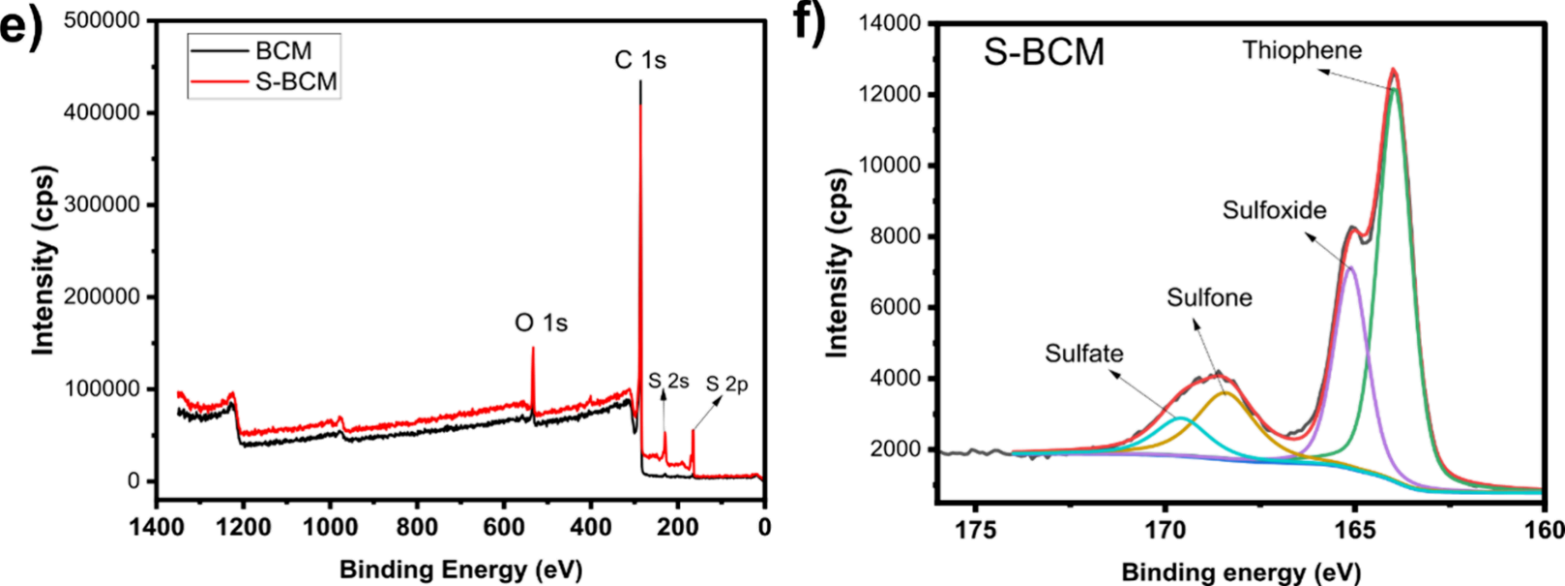
SEM morphological images of (c) BCM and (d) S-BCM samples. (e) XPS survey spectra of BCM and S-BCM samples. (f) Deconvoluted S 2p peaks of the S-BCM sample.

The discussion of panels c and d remains the same as the original version since they led to the same outcome: the doping process yields a sample with a rougher morphology.

The discussion of panels e and f remains the same as the original version since the same peaks and sulfur states were observed. However, the concentrations of the elements in the samples slightly changed. In the original article, the values of carbon, hydrogen, oxygen, and sulfur (in atomic ratio) were 88.1%, 0.98%, 3.5%, and 0.37%, respectively, for BCM and 84.7%, 1.2%, 4.6%, and 7.40% for S-BCM, respectively. The concentrations are now 92.7% (carbon), 5.2% (oxygen), and 0.7% (sulfur) for BCM and 82.7% (carbon), 8.2% (oxygen), and 7.1% (sulfur) for S-BCM. These differences have no effect on the overall discussion and conclusions since both situations led to the same outcome: the doped sample has more sulfur than the nondoped sample.

Also, the Supporting Information should have included the following information about the FTIR and XRD analyses: Fourier transform infrared spectroscopy analysis (FTIR) was performed with a Bruker Vertex v80 instrument equipped with a Harrick Praying Mantis DRIFT cell, covering a spectral range from 400 to 4000 cm^–1^. The amorphous/crystalline phases of the carbon materials were analyzed by a Rigaku SmartLab 9 kW X-ray diffractometer, operating at 40 kV and 135 mA at a 2θ angle of 10–70° using Co as the source. The software for analysis was PDXL2 coming together with the XRD device.

